# Multisystem Inflammatory Syndrome (MIS) following SARS-CoV-2 vaccinations; a systematic review

**DOI:** 10.1186/s40794-023-00204-x

**Published:** 2023-11-05

**Authors:** Mohamed Elsaid, Arvind Nune, Deyaa Hesham, Fatma Mohamed Fouad, Hamsa Hassan, Heba Hamouda, Huda Sherif, Maya Magdy Abdelwahab, Nourelhoda Hegazi, Yasmena Abd El-Rahman

**Affiliations:** 1 Faculty of Medicine, 6Th of October, Misr University for Science and Technology, Giza, Egypt; 2 Medical Research Platform, Giza, Egypt; 3 Department of Rheumatology and General Medicine, Southport and Ormskirk Hospital NHS Trust, Southport, UK; 4 Faculty of Pharmacy, Ain Shams University, Cairo, Egypt; 5 Faculty of Science, Cairo University, Giza, Egypt; 6 Biology Department, College of Science, Sultan Qaboos University, Muscat, Oman; 7 Faculty of Pharmacy, Mansoura University, Mansoura, Egypt; 8 Faculty of Medicine, Menoufia University, Menoufia, Egypt; 9 Faculty of Medicine, Helwan University, Cairo, Egypt; 10 Faculty of Medicine, Alexandria University, Alexandria, Egypt; 11 Faculty of Medicine, Portsaid University, Portsaid, Egypt

**Keywords:** Multisystem inflammatory syndrome, COVID-19, SARS-CoV-2, COVID-19 vaccine, Vaccination, Systematic review

## Abstract

**Background:**

Although SARS-CoV-2 vaccines are generally safe, there are growing concerns about their link to a potentially life-threatening multi-system inflammatory syndrome following vaccination (MIS-V). We conducted this systematic review to elucidate the prevalence of MIS, severity, treatment, and outcomes following SARS-CoV-2 vaccination.

**Methods:**

We searched PubMed, Scopus, ScienceDirect, Google Scholar, Virtual Health Library (VHL), Cochrane Library, and Web of Science databases for articles and case reports about MIS-V. We performed a qualitative analysis of individual cases from the included studies.

**Results:**

Of the 1366 studies identified by database search, we retrieved twenty-six case reports and two cohort studies. We analyzed the data of 37 individual cases extracted from 27 articles. The average age of the cases included in this review was 18 (1–67) years, with the most being male (M: F 3.1:1). Of the 37 included cases, the cardiovascular system was the most affected system by MIS (36, 97.3%), followed by the gastrointestinal tract (32, 86.5%).

**Conclusion:**

MIS after SARS-CoV-2 vaccinations can be fatal, but the incidence is low. Prompt recognition of MIS and ruling out the mimickers are critical in the patient’s early recovery.

**Supplementary Information:**

The online version contains supplementary material available at 10.1186/s40794-023-00204-x.

## Introduction

Since December 2020, 13.41 billion doses of COVID-19 vaccines have been administered globally [1]. SARS-CoV-2 vaccines are generally safe and play a critical role in disease prevention by reducing the severity and spread of COVID-19 [2, 3]. However, adverse events following immunization (AEFI) have been reported following the administration of COVID-19 vaccines, with a reporting rate of 60.4 per 100,000 doses administered, according to a recent surveillance report from Ontario [4]. Although the AEFI caused by SARS-CoV-2 vaccines are typically mild [5], severe adverse events, including multisystem inflammatory syndrome (MIS), myocarditis, and thromboses with thrombocytopenia, have been occasionally reported [6].

MIS is a hyper-inflammatory condition affecting multiple organs and causing shock. In April 2020, it was primarily recognized among children in the United Kingdom (UK) [7]. Even though children usually experienced mild or asymptomatic SARS-CoV-2 infection compared to adults, MIS was recognized as a life-threatening complication of SARS-CoV-2 infection [7]. In children, MIS-C was diagnosed if infection persisted for 4–6 weeks despite mild or asymptomatic infection [8, 9]. The clinical manifestations of MIS-C were similar to diseases such as Kawasaki Disease (KD), Macrophage Activation Syndrome (MAS), and Toxic Shock Syndrome (TSS) [9]. A similar medical condition known as a multisystem inflammatory syndrome in adults (MIS-A) has been reported as an uncommon complication of SARS-CoV-2 infection in adults [10, 11]. The significance of MIS stems from the severity of its symptoms and uncertainty about its management. Recent cohort studies in the USA and Italy indicated an MIS-C incidence of 5.1 per 1,000,000 person-month in the USA and a cumulative incidence of 3.27 per 100,000 residents in Italy [12, 13].

MIS following SARS-CoV-2 vaccinations began to emerge in January 2021, much like MIS-C and MIS-A [14, 15]. Initial cases of MIS following SARS-CoV-2 vaccinations had preceded SARS-CoV-2 infection [14], but later cases of MIS following SARS-CoV-2 vaccination in patients without prior SARS-CoV-2 infection were reported [15]. This new type of MIS was termed MIS-V. However, little is known about MIS-V, particularly its prevalence, treatment, and prognosis. This systematic review (SR) was carried out in adults and children to assess the prevalence, clinical features, treatment, and outcomes of people who developed MIS after receiving SARS-CoV-2 vaccinations.

## Methods

### Study design

This systematic review was conducted according to the PRISMA guidelines. The protocol was registered in PROSPERO (CRD42022314973). PRISMA checklist for the abstract and manuscript are provided in (Supplementary Tables 1 and 2).

### Search strategy

We searched PubMed, Scopus, ScienceDirect, Google Scholar, Virtual Health Library (VHL), Cochrane Library, and Web of Science (WOS) databases to include the studies in any language published on or before March 2nd, 2022. Additionally, we performed a manual search to include studies that were not retrieved through the database search. A manual search was done by screening the references of included studies, the cited articles in Google Scholar and similar articles in PubMed.

### Selection criteria

We chose the articles based on the following inclusion criteria (1) Adults, or children who received any SARS-CoV-2 vaccination (2) Developed multisystem inflammatory syndrome after SARS-CoV-2 vaccination (3) Original primary study design. We used combinations of the search words (‘’Multisystem inflammatory syndrome’’ or MIS or MIS-C or MIS-A or MIS-V) and (vaccine or vaccination or vaccines or postvaccination or postvaccine). We excluded review articles, duplicates, and studies with only an abstract or unavailable full text.

### Screening

**Fig. 1:**
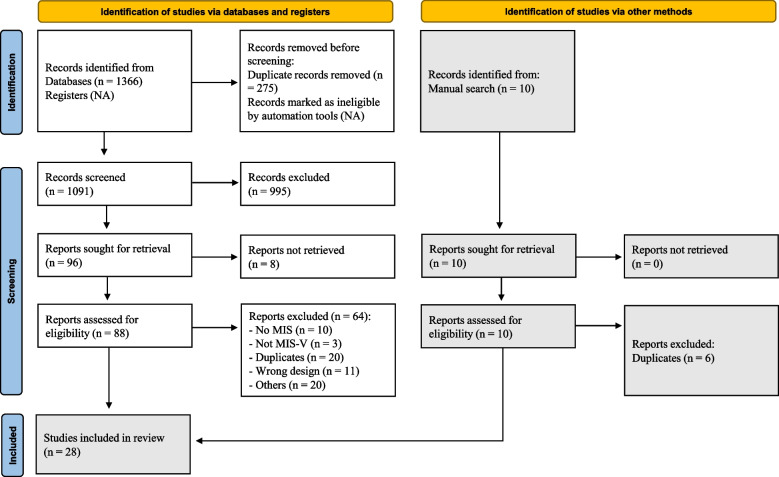
PRISMA flow diagram of the included studies

Two reviewers screened the articles’ titles and abstracts, and any differences were discussed. Then, full-text screening was performed in the same manner as title and abstract screening, with two independent authors responsible for each study, and the contrasts were discussed. In the event of a disagreement, a senior author was consulted to reach a consensus. The literature screening is further detailed in (Fig. 1).

### Data extraction

Two reviewers were primarily responsible for data extraction from full-text articles that met our selection criteria. The extracted data included case and vaccination characteristics, adverse effects based on the system, MIS diagnosis, hospitalization time, treatment, and follow-up. A cross-checking process was used to ensure accuracy, and all differences were discussed.

### Risk of bias assessment

All reviewers assessed the risk of bias by using the Joanna Briggs Institute reviewers’ manual (JBI) [16], and any disagreements were resolved through discussion. Each question’s answer could be one of the following: “Yes, No, Not applicable, Unclear”. If the answer was Yes, one point was awarded. Otherwise, it received a zero. We classified the assessment into three levels based on the final score of all questions: good (6–8 points), fair (3–5 points), and bad (0–2 points).

### Data analysis

Data were extracted into an Excel sheet and analyzed qualitatively. The MIS cases were defined based on the temporal association with COVID-19 vaccination due to the absence of a universally-agreed definition. MIS-V was further classified into probable or definite. A definite MIS-V case was defined when it developed following vaccination with no preceding evidence of COVID-19 infection or where the authors explicitly stated that MIS was due to vaccination. On the other hand, MIS-V was considered probable when there was evidence of prior COVID-19 vaccination and probable infection. Additionally, patients' demographic and clinical data were summarized and analyzed qualitatively.

## Results

### Search results

Initially, we identified 1366 papers from the databases. After removing 275 duplicates, we conducted title-abstract screening for the remaining 1091 articles and excluded 995 for not containing relevant data about MIS after SARS-CoV-2 vaccination or for inappropriate study design. The number of results for each database is provided in (Supplementary Table 3). We performed full-text screening on 96 articles and excluded 64 reports because they did not meet our inclusion criteria (Fig. 1), while eight more were not retrieved. Furthermore, we included four articles from manually searching the reference lists of the already included articles, making 28 articles in total.

### Study characteristics

We included 26 case reports [15, 17–41] presenting 28 individual cases, as well as a prospective cohort study [42] investigating nine patients who developed MIS after receiving SARS-CoV-2 vaccination. Of these 37 cases, 19 were children, while 18 were adults. Additionally, one cohort study was conducted in France on 33 pediatric participants diagnosed with MIS. Among them, seven cases developed MIS after administration of the first dose of the SARS-CoV-2 vaccine [43]. More details of included articles’ characteristics are shown in (Table 1**).**

**Table 1:** Individual case characteristics included in this SR

Case reports study characteristics					
**Study**	Country	Age (years)	Gender	Vaccine	Survival
					1 = Survive, 0 = Death
**Joshi et al. ** [17]	India	19	F	NR	0
**Yalçinkaya et al. ** [18]	Turkey	12	M	mRNA vaccine (Pfizer-BioNTech)	1
**DeJong et al. ** [19]	USA	14	F	Pfizer vaccine	1
**Abdelgalil et al. ** [20]	Saudi Arabia	12	M	Moderna vaccine	1
**McGann et al. ** [21]	New Zealand	16	M	Pfizer-BioNTech mRNA SARS-CoV-2	1
**Miyazato et al. ** [22]	Japan	32	M	BNT162b2(Pfizer-BioNTech	1
**Choi et al. ** [23]	South Korea	22	F	ChAdOx1 COVID-19 vaccine (AstraZeneca	1
**Bova et al. ** [24]	Italy	46	M	Ad26. COV2. S vaccine (Johnson &Johnson/Janssen)	1
**Chai et al. ** [25]	Denmark	17	M	Pfizer-BioNTech	1
**Poussaint et al. ** [26]	USA	12	M	Pfizer-BioNTech	1
**Lee et al. ** [27]	United States	13	M	Pfizer/BioNTech vaccine (BNT162b2)	1
**Al Bishawi et al. ** [28]	Qatar	37	F	Modern mRNA vaccine	1
**Park et al. ** [29]	Korea	67	M	AstraZeneca vaccine / ChAdOx1 nCoV-19	1
**Buchhorn et al. ** [30]	Germany	18	M	Pfizer/BionTech (BNT162b2)	1
**Grome et al. ** [41]	USA	in 30 s	M	Pfizer/BioNTech mRNA COVID-19 vaccine	0
**Nune et al. ** [15]	UK	44	F	Pfizer-BioNTech mRNA vaccine	1
**Uwaydah et al. ** [31]	United Arab Emirates	22	M	inactivated SARS-CoV-2 vaccine	1
**Agarwal et al. ** [32]	India	28	M	NR	1
**Bangash et al. ** [33]	USA	21	F	J&J COVID-19 vaccine	1
**Deb et al. ** [34]	USA	67	M	Moderna COVID-19 vaccine	1
**Koga et al. ** [36]	Japan	15	M	m RNA (Moderna)	1
**Lieu et al. ** [37]	Canada	21	F	m RNA (Moderna)	NR
**Baicus et al. ** [38]	Romania	22	M	BNT162b2	1
**Kahn et al. ** [39]	USA	20	M	(Pfizer-BioNTech)	1
**Taneja et al. ** [40]	India	27	M		1
**Salzman et al. ** [35]	USA	20	F	Pfizer-BioNTech	1
		40	M		1
		18	M		1
**Ouldali et al. ** [42]	France	12–17	M	BNT162b2 vaccine (mRNA vaccine)	1
			M		1
			M		1
			M		1
			M		1
			M		1
			F		1
			M		1
			M		1

*MIS* Multisystem inflammatory syndrome, *USA* The U States of America, *UK* The United Kingdom, *F* Female, *M* Male, *COVID-19* Coronavirus disease 2019, *NR* Not Reported

### Risk of bias assessment

The JBI quality assessment showed that 15 articles demonstrating case reports were good quality, while 11 articles were fair quality (Supplementary Table 4). The two cohort studies showed good and fair scores, as shown in (Supplementary Table 5).

### Qualitative analysis

#### Patients’ characteristics

The median age of patients included in this review was 18 (1–67) years, most being male (M: F 3.1:1). The age group most represented was the pediatric population, accounting for 51.4% of reported MIS cases. Most patients were from the United States (10, 27%), followed by France (9, 24.3%) and India (3, 8.1%). Fourteen patients (37%) presented with a chronic condition before vaccination, with the most prevalently reported condition being asthma (4, 10.8%). An overview of all chronic diseases reported is shown in (Table 2).

**Table 2:** Summary of cases characteristics

Variable	Total (*n* = 37)
**Age (years) Median (Range)**	18 (1–67)
**Gender M: F**	3.1:1
**Country n (%)**	
Canada	1 (2.7)
Germany	1 (2.7)
India	3 (8.1)
Italy	1 (2.7)
Japan	2 (5.4)
Korea	1 (2.7)
New Zealand	1 (2.7)
Qatar	1 (2.7)
South Korea	1 (2.7)
Turkey	1 (2.7)
United Arab Emirates	1 (2.7)
United Kingdom	2 (5.4)
United States	10 (27.0)
France	9 (24.3)
Romania	1 (2.7)
Denmark	1 (2.7)
**Age Group n (%)**	
Paediatric	19 (51.4)
Adult	18 (48.6)
**Chronic Disease n (%)**	
Haemoglobin SS disease	1 (2.7)
Methicillin sensitive staphylococcal septic arthritis	1 (2.7)
Lyme disease	1 (2.7)
Hypertension	2 (5.4)
Diabetes	2 (5.4)
Hypoxic ischemic encephalopathy	1 (2.7)
Epilepsy	1 (2.7)
Asthma	4 (10.8)
Depression	1 (2.7)
Hyperlipidaemia	1 (2.7)
Osteochondritis	1 (2.7)
Leukaemia	1 (2.7)

#### Vaccine status

All 37 patients across the twenty-eight included articles had received at least one SARS-CoV-2 vaccine dose, with the most commonly administered vaccines being Pfizer-BioNTech (24, 64.9%) and Moderna (5, 13.5%). One patient received the Moderna vaccination as a first dose and the Pfizer-BioNTech vaccination as a second dose. Following vaccination, most patients did not contract COVID-19 (67.6%), with 7.1% infected after vaccination (Table 3).

**Table 3:** Vaccine status and incidence of COVID-19 post-vaccination

Variable	Total (*n* = 37)
**Number of doses before having MIS**	
One dose	20 (54.1)
Two doses	16 (43.2)
Not reported	1 (2.7)
**Name of vaccine**	
Pfizer-BioNTech	24 (64.9)
AstraZeneca	2 (2.7)
Moderna	5 (13.5)
Johnson and Johnson	2 (5.4)
Inactivated SARS-CoV-2 vaccine	1 (2.7)
Name not recorded	3 (8.1)
**COVID-19 infection post-vaccine**	
Yes	4 (10.8)
No	25 (67.6)
Not reported	8 (21.6)
**COVID-19 exposure post-vaccine**	
Yes	2 (5.4)
No	8 (21.6)
Not reported	27 (73.0)

*MIS* Multisystem inflammatory syndrome, *SARS-COV-2* Severe acute respiratory syndrome coronavirus 2, *COVID-19* Coronavirus disease 2019

#### MIS presentation and patient outcomes

All patients discussed presented with MIS-V, spanning 19 (51.4%) paediatric patients and 18 (45.9%) adult patients. In total, 29 cases had a positive COVID-19 test either by PCR, anti-spike or anti-nucleocapsid antibody test or combination. However, only 8 cases were positive using PCR. The remaining three cases had positive results both before and after vaccination. In all patients, MIS was suspected to result from the SARS-CoV-2 vaccination (MIS-V), where 23 were definite MIS-V and only 14 were probable. However, the criteria for MIS diagnosis differed between the included data, with eight cases depending on CDC criteria while 9 cases used the WHO criteria. The average onset of MIS was 16.4 days post-previous vaccination dose, and 24 patients (64%) were infection naïve when they developed MIS, indicative of definitive MIS-V.

All systems were affected except the endocrine and reproductive systems. Out of 37 patients, 36 (97.3%) of the cases experienced cardiovascular symptoms such as myocarditis, tachycardia, hypertension, and pericardial effusion. In comparison, 32 cases (86.5%) experienced gastrointestinal complications such as vomiting, abdominal pain, and diarrhoea, and 25 (67.6%) of the cases experienced immune system complications such as inflammation, lymphopenia, and fever, as well as circulatory system complications such as hypotension, coagulopathy, and tachycardia.

All patients included in this review were hospitalized, and the average duration was 16.8 days. A total of 14 patients (37.9%) were admitted to the intensive care unit for an average duration of six days, and corticosteroids were the primary treatment used (30, 81.1%) (Table 4). Most patients recovered (91.9%), and (2, 8.1%) died. Of the two patients that did not survive, one was a young adult who developed bradycardia and asystole [17], and the second was an adult that developed multi-organ failure [41]. The mean follow-up duration post-hospital admission was 26.5 days.

**Table 4:** Multisystem inflammatory syndrome by organ involvement, management and patient outcomes

Variable	Total (*n* = 37)
**Adverse Effects by system**	
CNS	21 (56.8)
CVS	36 (97.3)
GIT	32 (86.5)
Respiratory	20 (54.1)
Skeletal	16 (43.2)
Urinary	12 (32.4)
Endocrine	0 (0)
Reproductive	0 (0)
Circulatory	25 (67.6)
Lymphatic	12 (32.4)
Integumentary	21 (56.8)
Immune	25 (67.6)
**Laboratory findings**	
Positive SARS CoV-2 anti nucleoplasmid	14 (37.8)
Positive Anti spike antibodies	20 (54.1)
Positive SARS-CoV-2 antigen	14 (37.8)
Raised acute phase reactants	33 (88.2)
**Treatment**	
IVIG	24 (64.9)
Steroids	30 (81.1)
Other treatment	26 (70.3)
**Outcomes**	
Survived	34 (91.9)
Hospitalization	36 (100)
Duration (days)	16.8 (4–90)
Admitted to ICU	14 (37.9)
Duration (days)	6
**Follow-up duration (days)**	26.5 (0–90)

*SARS-COV-2* Severe acute respiratory syndrome coronavirus 2, *CNS* Central nervous system, *CVS* Cardiovascular system, *GIT* Gastrointestinal tract, *IVIG* Intravenous Immunoglobulin, *ICU* Intensive care unit

#### Laboratory findings

Laboratory findings identified acute phase reactants in 33 patients (88.2%) and anti-spike antibodies in 20 patients (54.1%) prior to vaccination. Additionally, SARS-CoV-2 anti-nucleoplasmid was observed in 14 (37.8%) cases (Table 4). Although SARS-CoV-2 antigen was positive in 14 patients, 4 cases had negative PCR/Antibody tests.

## Discussion

Our SR discovered that several patients who developed post-vaccination MIS had no prior SARS-CoV-2 infection. It was also noted that mortality from MIS was low, and recovery was high after appropriate therapies. In addition, acute-phase reactants were elevated in all patients with a suspected MIS-V. Acute phase reactants comprise c-reactive protein (CRP), ferritin, fibrinogen, hepcidin, and serum amyloid-A are crucial markers of inflammation [44]. Therefore, the raised inflammatory markers with multiorgan involvement support a diagnosis of MIS.

MIS is a rare but severe complication of a SARS-CoV-2 infection that typically arises between two and twelve weeks following the initial infection. The definition of MIS-V, however, differs depending on the patient’s age. As per the Brighton Collaboration network recommendation, MIS-V is diagnosed in children if patients experience the onset of MIS symptoms within a four-to-six-week period and in adults for up to two to twelve weeks after SARS-CoV-2 vaccination [11]. However, given the novel nature of MIS-V, it is possible to diagnose it in case clinical symptoms of MIS are present outside of this window. Given the rarity of MIS-V, the exact incidence, prevalence, and pathophysiology are poorly comprehended. However, there have been several proposed hypotheses, including dysregulation of the immune system, cytokine storm or hyperreactivity of the immune system [35].

During the COVID-19 pandemic, MIS was primarily reported in children (MIS-C) and later reported in adults (MIS-A). MIS-C has various clinical features, including shock, abdominal pain, cardiac dysfunction, myocarditis, and elevated inflammatory markers [46}. On the other hand, the typical presentation of MIS-A contains fever over 24 h, severe cardiac illness, rash, non-purulent conjunctivitis, and elevated inflammatory markers. It was described as a severe extra-pulmonary multiorgan dysfunction associated with circulatory shock without severe respiratory disease [37, 45]. Aligning with previous studies, the extra-pulmonary multiorgan involvement was reflected in this review, with cardiovascular events being the most prevalently observed. SARS-CoV-2 vaccine adverse effects on the cardiovascular system have been reported. Studies have found that the SARS-CoV-2 vaccine was associated with increased myocarditis cases. More studies reported a rare occurrence of MIS‐A and MIS-C myocarditis associated with SARS-CoV-2 vaccines [46, 47]. However, cardiac complications related to SARS-CoV-2 infection and MIS post-COVID-19 were more severe than SARS-CoV-2 vaccine-associated myocarditis [47].

Vogel et al. described the need to define MIS-V as an adverse event following immunization against SARS-CoV-2, including infection naïve patients. In this SR, we observed eight patients who developed MIS-V without evidence of a prior SARS-CoV-2 infection either by PCR, anti-nucleocapsid, or anti-spike antibodies. However, notable differences were observed between the characteristics, outcomes, and mortality rate of infection naïve MIS-V and MIS-V with prior infection. There are three possible ways of developing MIS-V: (1) MIS following SARS-CoV-2 vaccination without prior SARS-CoV-2 infection, which can be directly attributed to vaccination; (2) MIS in those who have had COVID-19 and are then vaccinated; (3) MIS in those without prior SARS-CoV-2 infection before vaccination and then contract COVID-19 before presenting with an illness resembling MIS [11]. Unfortunately, the limited data available in the literature on the characteristic presentation of these three MIS-V routes compromises a viable comparison. Grome et al. reported a fatal case of MIS-A following complete SARS-CoV-2 vaccination with prior SARS-CoV-2 infection six weeks before [3]. Moreover, Jain et al. reported two successfully treated cases of MIS-C occurring after SARS-CoV-2 vaccination, with one case with a history of prior SARS-CoV-2 infection, suggesting that the SARS-CoV-2 vaccine might have modulated the pathogenesis of those MIS-C cases with different routes [4]. Similar cases of MIS development after receiving SARS-CoV-2 vaccines with prior community-acquired COVID-19 were documented [33], further highlighting the vaccine’s involvement in MIS [44]. Moreover, few reports discussed the COVID-19 exposure of patients’ post-vaccination; hence, a thorough assessment of the relationship between exposure and contracting an infection could not be explored in the included articles. The infection must be ruled out before arriving at a definitive MIS diagnosis. Therefore, a confirmed MIS-V diagnosis was not made in some cases reviewed here because the infection could not be ruled out. Notwithstanding, as shown by the work of Nune et al., patients could present with clinical signs of infection yet not have an infection; in such cases, clinical tests can rule out Covid-19 or bacterial infection and aid diagnosis of MIS [12].

A study published in the Lancet revealed that children of male sex, aged 5–11 years, with foreign-born parents, asthma, obesity, and a life-limiting condition were more likely to develop MIS. Studies have shown that COVID-19 vaccination is associated with reduced incidence of MIS in children, especially if 2 doses are given. A study of MIS cases in France during September–October 2021 found a significantly lower risk of MIS among vaccinated adolescents than those unvaccinated [48]. Zambrano et al. found a 91% protective effect of complete (2 doses) BNT162b2 vaccination against MIS in children [49]. Phase 2 and phase 3 clinical trials of BNT162b2 revealed no cases of MIS-C after vaccination [43]. Despite the reports of post-vaccination MIS, vaccination clearly lowers the overall MIS burden, probably by preventing infection. These studies also suggest a low likelihood of vaccination triggering the development of MIS.

MIS patients are treated with different treatment regimens, mostly revolving around the use of glucocorticoids, and immunomodulatory medications, including intravenous immune globulin (IVIG), as first-tier therapy. The patients who are refractory to glucocorticoids and IVIG warrants a step-up of immunomodulatory therapy to therapeutic plasmapheresis [50] and biological agents such as anakinra, tocilizumab, and infliximab [51].

Currently, there are several guidelines and recommendations for managing MIS in children associated with COVID-19 [52]. Nearly, all of them recommend the administration of combinations of corticosteroids (oral or IV methylprednisolone), immunomodulatory therapy (e.g., IVIG), and anti-thrombotic treatment (i.e., aspirin). According to the American Academy of Paediatrics, antibiotic treatment and fluid resuscitation are recommended when the patients are hypotensive or appear to be in septic shock. IVIG is recommended to use at 2 gm/Kg. Refractory cases or those requiring ICU admission are treated with 2 to 30 mg/Kg/day IV methylprednisolone, and biological treatment (i.e., anakinra 2 to 10 mg/Kg/day). Also, low-dose aspirin should be initiated as thromboprophylaxis unless contraindicated (e.g., platelets < 100.000). Generally, a multidisciplinary clinical team, including a rheumatologist, cardiologist, critical care, and infectious disease specialists, should be consulted for an individualized treatment plan based on the case presentation [53]. Like MIS management in children, the National Institutes of Health (NIH) COVID-19 guidelines and the American College of Rheumatology described similar recommendations for MIS in adults [8].

Our SR has a few limitations. The level of evidence might be low due to case reports being included. Due to the reporting bias, we might have missed some of the investigation results. However, strict inclusion and exclusion criteria and a thorough analysis of all included articles by multiple researchers minimized the bias. Because of the small number of reported cases, comparing the characteristics and severity of MIS-V across the nations and in various ethnic groups was not possible.

## Conclusion

MIS following SARS-CoV-2 vaccinations is rare, indicating that vaccinations against SARS-CoV-2 infection are generally safe and well tolerated. This review showed that most patients who developed MIS following SARS-CoV-2 vaccinations had no prior SARS-CoV-2 infection, and the MIS-V mortality was low. Most patients recovered from MIS-V after receiving immunosuppressive medications such as corticosteroids and immunoglobulins. Raising awareness, prompt recognition of MIS-V, and ruling out sepsis mimicking MIS are vital for patients’ survival and early recovery.

### Supplementary Information

**Additional file 1: Supplementary Table 1.** PRISMA 2020 checklist for abstract. **Supplementary Table 2.** PRISMA 2020 checklist for systematic review and meta-analysis. **Supplementary Table 3.** Database search results. **Supplementary Table 4.** Quality assessment table for case reports. **Supplementary Table 5.** Quality assessment table for cohort studies.

## Data Availability

Data will be available upon request from the corresponding author.
