# Supplementary material for: Multisystem Inflammatory Syndrome (MIS) following SARS-CoV-2 vaccinations; a systematic review
Source: Trop Dis Travel Med Vaccines. 2023 Nov 5;9:19. doi: 10.1186/s40794-023-00204-x (PMC10625711; doi:10.1186/s40794-023-00204-x)
Supplement: Supplementary file 1 — Additional file 1: Supplementary Table 1. PRISMA 2020 checklist for abstract. Supplementary Table 2. PRISMA 2020 checklist for systematic review and meta-analysis. Supplementary Table 3. Database search results. Supplementary Table 4. Quality assessment table for case reports. Supplementary Table 5. Quality assessment table for cohort studies. [file 40794_2023_204_MOESM1_ESM.docx]

# Supplementary Table 1. PRISMA 2020 checklist for abstract

| **Section and Topic** | **Item #** | **Checklist item** | **Reported (Yes/No)** |  |  |
| --- | --- | --- | --- | --- | --- |
| **TITLE** | | |  |  |  |
| Title | 1 | Identify the report as a systematic review. | Yes |  |  |
| **BACKGROUND** | | |  |  |  |
| Objectives | 2 | Provide an explicit statement of the main objective(s) or question(s) the review addresses. | Yes |  |  |
| **METHODS** | | |  |  |  |
| Eligibility criteria | 3 | Specify the inclusion and exclusion criteria for the review. | Yes |  |  |
| Information sources | 4 | Specify the information sources (e.g. databases, registers) used to identify studies and the date when each was last searched. | Yes |  |  |
| Risk of bias | 5 | Specify the methods used to assess risk of bias in the included studies. | Yes |  |  |
| Synthesis of results | 6 | Specify the methods used to present and synthesise results. | Yes |  |  |
| **RESULTS** | | |  |  |  |
| Included studies | 7 | Give the total number of included studies and participants and summarise relevant characteristics of studies. | Yes |  |  |
| Synthesis of results | 8 | Present results for main outcomes, preferably indicating the number of included studies and participants for each. If meta-analysis was done, report the summary estimate and confidence/credible interval. If comparing groups, indicate the direction of the effect (i.e. which group is favoured). | Yes |  |  |
| **DISCUSSION** | | |  |  |  |
| Limitations of evidence | 9 | Provide a brief summary of the limitations of the evidence included in the review (e.g. study risk of bias, inconsistency and imprecision). | No |  |  |
| Interpretation | 10 | Provide a general interpretation of the results and important implications. | Yes |  |  |
| **OTHER** | | |  |  |  |
| Funding | 11 | Specify the primary source of funding for the review. | NA |  |  |
| Registration | 12 | Provide the register name and registration number. | NA |  |  |

# Supplementary Table 2. PRISMA 2020 checklist for systematic review and meta-analysis

| **Section and Topic** | **Item #** | **Checklist item** | **Location where item is reported** |  |  |
| --- | --- | --- | --- | --- | --- |
| **TITLE** | | |  |  |  |
| Title | 1 | Identify the report as a systematic review. | 1 |  |  |
| **ABSTRACT** | | |  |  |  |
| Abstract | 2 | See the PRISMA 2020 for Abstracts checklist. | 2, 3 |  |  |
| **INTRODUCTION** | | |  |  |  |
| Rationale | 3 | Describe the rationale for the review in the context of existing knowledge. | 3, 4 |  |  |
| Objectives | 4 | Provide an explicit statement of the objective(s) or question(s) the review addresses. | 4 |  |  |
| **METHODS** | | |  |  |  |
| Eligibility criteria | 5 | Specify the inclusion and exclusion criteria for the review and how studies were grouped for the syntheses. | 5 |  |  |
| Information sources | 6 | Specify all databases, registers, websites, organisations, reference lists and other sources searched or consulted to identify studies. Specify the date when each source was last searched or consulted. | 5 |  |  |
| Search strategy | 7 | Present the full search strategies for all databases, registers and websites, including any filters and limits used. | Supplementary table 3 |  |  |
| Selection process | 8 | Specify the methods used to decide whether a study met the inclusion criteria of the review, including how many reviewers screened each record and each report retrieved, whether they worked independently, and if applicable, details of automation tools used in the process. | 5 |  |  |
| Data collection process | 9 | Specify the methods used to collect data from reports, including how many reviewers collected data from each report, whether they worked independently, any processes for obtaining or confirming data from study investigators, and if applicable, details of automation tools used in the process. | 6 |  |  |
| Data items | 10a | List and define all outcomes for which data were sought. Specify whether all results that were compatible with each outcome domain in each study were sought (e.g. for all measures, time points, analyses), and if not, the methods used to decide which results to collect. | 6 |  |  |
|  | 10b | List and define all other variables for which data were sought (e.g. participant and intervention characteristics, funding sources). Describe any assumptions made about any missing or unclear information. | 6 |  |  |
| Study risk of bias assessment | 11 | Specify the methods used to assess risk of bias in the included studies, including details of the tool(s) used, how many reviewers assessed each study and whether they worked independently, and if applicable, details of automation tools used in the process. | 6 |  |  |
| Effect measures | 12 | Specify for each outcome the effect measure(s) (e.g. risk ratio, mean difference) used in the synthesis or presentation of results. | NA |  |  |
| Synthesis methods | 13a | Describe the processes used to decide which studies were eligible for each synthesis (e.g. tabulating the study intervention characteristics and comparing against the planned groups for each synthesis (item #5)). | 6 |  |  |
|  | 13b | Describe any methods required to prepare the data for presentation or synthesis, such as handling of missing summary statistics, or data conversions. | 6 |  |  |
|  | 13c | Describe any methods used to tabulate or visually display results of individual studies and syntheses. | 6 |  |  |
|  | 13d | Describe any methods used to synthesize results and provide a rationale for the choice(s). If meta-analysis was performed, describe the model(s), method(s) to identify the presence and extent of statistical heterogeneity, and software package(s) used. | NA |  |  |
|  | 13e | Describe any methods used to explore possible causes of heterogeneity among study results (e.g. subgroup analysis, meta-regression). | NA |  |  |
|  | 13f | Describe any sensitivity analyses conducted to assess robustness of the synthesized results. | NA |  |  |
| Reporting bias assessment | 14 | Describe any methods used to assess risk of bias due to missing results in a synthesis (arising from reporting biases). | 6 |  |  |
| Certainty assessment | 15 | Describe any methods used to assess certainty (or confidence) in the body of evidence for an outcome. | NA |  |  |
| **RESULTS** | | |  |  |  |
| Study selection | 16a | Describe the results of the search and selection process, from the number of records identified in the search to the number of studies included in the review, ideally using a flow diagram. | 6, 7 |  |  |
|  | 16b | Cite studies that might appear to meet the inclusion criteria, but which were excluded, and explain why they were excluded. | NA |  |  |
| Study characteristics | 17 | Cite each included study and present its characteristics. | 7 |  |  |
| Risk of bias in studies | 18 | Present assessments of risk of bias for each included study. | 7 |  |  |
| Results of individual studies | 19 | For all outcomes, present, for each study: (a) summary statistics for each group (where appropriate) and (b) an effect estimate and its precision (e.g. confidence/credible interval), ideally using structured tables or plots. | 7, 8, 9, 10 |  |  |
| Results of syntheses | 20a | For each synthesis, briefly summarise the characteristics and risk of bias among contributing studies. | 7 |  |  |
|  | 20b | Present results of all statistical syntheses conducted. If meta-analysis was done, present for each the summary estimate and its precision (e.g. confidence/credible interval) and measures of statistical heterogeneity. If comparing groups, describe the direction of the effect. | NA |  |  |
|  | 20c | Present results of all investigations of possible causes of heterogeneity among study results. | NA |  |  |
|  | 20d | Present results of all sensitivity analyses conducted to assess the robustness of the synthesized results. | NA |  |  |
| Reporting biases | 21 | Present assessments of risk of bias due to missing results (arising from reporting biases) for each synthesis assessed. | 7 |  |  |
| Certainty of evidence | 22 | Present assessments of certainty (or confidence) in the body of evidence for each outcome assessed. | 7 |  |  |
| **DISCUSSION** | | |  |  |  |
| Discussion | 23a | Provide a general interpretation of the results in the context of other evidence. | 10 |  |  |
|  | 23b | Discuss any limitations of the evidence included in the review. | 12 |  |  |
|  | 23c | Discuss any limitations of the review processes used. | 12 |  |  |
|  | 23d | Discuss implications of the results for practice, policy, and future research. | 12 |  |  |
| **OTHER INFORMATION** | | |  |  |  |
| Registration and protocol | 24a | Provide registration information for the review, including register name and registration number, or state that the review was not registered. | 5 |  |  |
|  | 24b | Indicate where the review protocol can be accessed, or state that a protocol was not prepared. | 5 |  |  |
|  | 24c | Describe and explain any amendments to information provided at registration or in the protocol. | NA |  |  |
| Support | 25 | Describe sources of financial or non-financial support for the review, and the role of the funders or sponsors in the review. | 13 |  |  |
| Competing interests | 26 | Declare any competing interests of review authors. | 13 |  |  |
| Availability of data, code and other materials | 27 | Report which of the following are publicly available and where they can be found: template data collection forms; data extracted from included studies; data used for all analyses; analytic code; any other materials used in the review. | 13 |  |  |

# Supplementary Table 3. Database search results

| Search string | Database or further sources | Results (N=1386) | Date | Comments |
| --- | --- | --- | --- | --- |
| ("Multisystem inflammatory syndrome" or MIS or MIS-C or MIS-A or MIS-V) and (vaccine or vaccination or vaccines or postvaccination or postvaccine) | PubMed | 211 | 2nd March 2022 |  |
| ("Multisystem inflammatory syndrome" or MIS or MIS-C or MIS-A or MIS-V) and (vaccine or vaccination or vaccines or postvaccination or postvaccine) | Scopus | 301 | 2nd March 2022 | TITLE-ABS-KEY |
| ("Multisystem inflammatory syndrome" OR MIS OR MIS-A OR MIS-C) AND (vaccine OR vaccination OR vaccines OR postvaccination OR postvaccine) | Science Direct | 318 | 2nd March 2022 | Title, abstract or author-specified keywords |
| ("Multisystem inflammatory syndrome" or MIS or MIS-C or MIS-A or MIS-V) and (vaccine or vaccination or vaccines or postvaccination or postvaccine) | WOS | 296 | 2nd March 2022 | All fields |
| ("Multisystem inflammatory syndrome" or MIS or MIS-C or MIS-A or MIS-V) and (vaccine or vaccination or vaccines or postvaccination or postvaccine) | VHL | 203 | 2nd March 2022 | Title, abstract, subject |
| ("Multisystem inflammatory syndrome" or MIS or MIS-C) and (vaccine or vaccination or vaccines or postvaccination or postvaccine) | Cochrane | 15 | 2nd March 2022 | All text |
| allintitle: vaccine OR vaccination OR vaccines OR postvaccination OR postvaccine "Multisystem inflammatory syndrome" | Google Scholar | 32 | 2nd March 2022 |  |
| allintitle: MIS-V vaccine OR vaccination OR vaccines OR postvaccination OR postvaccine |  | 4 |  |  |
| allintitle: MIS-A vaccine OR vaccination OR vaccines OR postvaccination OR postvaccine |  | 2 |  |  |
| allintitle: MIS-C vaccine OR vaccination OR vaccines OR postvaccination OR postvaccine |  | 4 |  |  |

# Supplementary Table 4. Quality assessment table for case reports

| Reference ID | 1 | 2 | 3 | 4 | 5 | 6 | 7 | 8 | Total | Score |
| --- | --- | --- | --- | --- | --- | --- | --- | --- | --- | --- |
| Joshi et al. 2022 | 1 | 0 | 1 | 1 | 1 | 1 | 1 | UC | 6 | Good |
| Yalçinkaya et al. 2022 | 1 | NA | 1 | 1 | 1 | 1 | 1 | 1 | 7 | Good |
| DeJong et al. 2022 | 1 | 0 | 1 | 1 | 0 | 1 | 0 | 1 | 5 | Fair |
| Abdelgalil et al. 2022 | 1 | 0 | 1 | 0 | 1 | 1 | 0 | 0 | 4 | Fair |
| McGann et al. 2022 | 1 | 1 | 1 | 1 | 1 | 1 | 1 | 1 | 8 | Good |
| Miyazato et al. 2022 | 1 | 0 | 1 | 0 | 0 | 1 | 0 | 0 | 3 | Fair |
| Choi et al. 2022 | 1 | 0 | 1 | 1 | 1 | 1 | 1 | 0 | 6 | Good |
| Bova et al. 2022 | 1 | 0 | 1 | 1 | 0 | 0 | 0 | 0 | 3 | Fair |
| Chai et al. 2021 | 1 | 0 | 1 | 1 | 0 | 0 | 0 | 0 | 3 | Fair |
| Poussaint et al. 2021 | 1 | 1 | 1 | 1 | 0 | 1 | 0 | 0 | 5 | Fair |
| Lee et al. 2021 | 1 | 1 | 1 | 1 | 0 | 1 | NA | 1 | 6 | Good |
| Al Bishawi et al. 2021 | 1 | 1 | 1 | 1 | 1 | 1 | NA | 1 | 7 | Good |
| Park et al. 2021 | 1 | 1 | 1 | 1 | 0 | 1 | NA | 1 | 6 | Good |
| Buchhorn et al. 2021 | 1 | 1 | 0 | 1 | 0 | 1 | NA | 1 | 5 | Fair |
| Grome et al. 2021 | 0 | 0 | 1 | 1 | 1 | 1 | NA | 1 | 5 | Fair |
| Nune et al. 2021 | 1 | 1 | 1 | 1 | 1 | 1 | NA | 1 | 7 | Good |
| Uwaydah et al. 2021 | 1 | 1 | 1 | 1 | 1 | 1 | NA | 1 | 7 | Good |
| Agarwal et al. 2021 | 1 | 0 | 1 | 1 | 1 | 1 | NA | 1 | 6 | Good |
| Bangash et al. 2021 | 0 | 0 | 1 | 1 | 0 | 1 | NA | 1 | 4 | Fair |
| Deb et al. 2021 | 1 | 0 | 1 | 1 | 1 | 1 | NA | 1 | 6 | Good |
| Koga et al. 2022 | 0 | 0 | 1 | 1 | 1 | 1 | UC | 1 | 5 | Fair |
| Lieu et al. 2022 | 1 | 0 | 1 | 1 | 1 | 1 | 1 | 1 | 7 | Good |
| Baicus et al. 2021 | 0 | 1 | 1 | 1 | 1 | 1 | 1 | 0 | 6 | Good |
| Kahn et al. 2021 | 1 | 0 | 1 | 1 | 1 | 1 | 0 | 1 | 6 | Good |
| Taneja V et al. 2021 | UC | 0 | 1 | 1 | 1 | UC | NA | 1 | 4 | Fair |
| Salzman et al. 2021 | 1 | 1 | 1 | 1 | 1 | 1 | NA | 1 | 7 | Good |

Yes = 1 No = 0 Not applicable = NA Unclear = UC

Score: Good = 6-8 Fair = 3-5 Bad = 0-2

1. Were patient’s demographic characteristics clearly described?

2. Was the patient’s history clearly described and presented as a timeline?

3. Was the current clinical condition of the patient on presentation clearly described?

4. Were diagnostic tests or methods and the results clearly described?

5. Was the intervention(s) or treatment procedure(s) clearly described?

6. Was the post-intervention clinical condition clearly described?

7. Were adverse events (harms) or unanticipated events identified and described?

8. Does the case report provide takeaway lessons?


# Supplementary Table 5. Quality assessment table for cohort studies

| Reference ID | 1 | 2 | 3 | | 4 | | 5 | | 6 | | 7 | 8 | | 9 | | 10 | | 11 | | Total | | | Score | |
| --- | --- | --- | --- | --- | --- | --- | --- | --- | --- | --- | --- | --- | --- | --- | --- | --- | --- | --- | --- | --- | --- | --- | --- | --- |
| Ouldali et al. 2022 | 1 | 1 | 1 | | UN | | UN | | 1 | | 1 | 1 | | UN | | UN | | 1 | | 7 | | | Fair | |
| Yousaf et al. 2022 | 1 | 1 | 1 | | 1 | | UN | | UN | | 1 | 1 | | 1 | | 1 | | 1 | | 9 | | | Good | |

Yes = 1 No = 0 Not applicable = NA Unclear = UC

Score: Good = 9-11 Fair = 4-8 Bad = 0-3

1. Were the two groups similar and recruited from the same population?

2. Were the exposures measured similarly to assign people to both exposed an unexposed group?

3. Was the exposure measured in a valid and reliable way?

4. Were confounding factors identified?

5. Were strategies to deal with confounding factors stated?

6. Were the groups/participants free of the outcome at the start of the study (or at the moment of exposure)?

7. Were the outcomes measured in a valid and reliable way?

8. Was the follow up time reported and sufficient to be long enough for outcomes to occur?

9. Was follow up complete, and if not, were the reasons to loss to follow up described and explored?

10. Were strategies to address incomplete follow up utilized?

11. Was appropriate statistical analysis used?
